# The Impact of Massed and Spaced-Out Curriculum in Oncology Knowledge Acquisition

**DOI:** 10.1007/s13187-017-1190-y

**Published:** 2017-02-13

**Authors:** Dario Cecilio-Fernandes, Wytze S. Aalders, Jakob de Vries, René A. Tio

**Affiliations:** 1 Center for Education Development and Research in Health Professions (CEDAR), University of Groningen and University Medical Center Groningen, Antonius Deusinglaan1, FC40, 9713 AV Groningen, The Netherlands; 2 University of Groningen, University Medical Center Groningen, Groningen, The Netherlands; 3 Department of Surgery, University of Groningen and University Medical Center Groningen, Groningen, The Netherlands; 4 Center for Education Development, Research in Health Professions (CEDAR) and Department of Cardiology, University of Groningen and University Medical Center Groningen, Groningen, The Netherlands

## Abstract

Starting in 2009, cancer has been the leading cause of death in the Netherlands. Oncology is therefore an important part of the medical curriculum in undergraduate education. It is crucial that medical students know about cancer, since doctors will encounter many cases of oncology. We have compared the influence that teaching oncology has when spread over a 3-year curriculum versus concentrated in one semester. The participants comprised 525 medical students from one medical school with comprehensive integrated curricula. Of those, 436 followed the massed curriculum, with oncology concentrated in one semester. The remaining 89 students followed a spaced-out curriculum, in which oncology was spread out over 3 years. To measure students’ knowledge, we used their progress test results from 2009 to 2012. All questions about oncology were categorized and selected. Because of our unbalanced sample and missing data and to reduce the chances for a type II error, we compared the growth of oncology questions using mixed effect models. A cubic growth model with an unstructured covariance matrix fitted our data best. At the start, students in the spaced-out curriculum scored higher on oncology questions. The initial growth was faster for the spaced-out curriculum students, whereas the acceleration over time was slower compared to the massed curriculum students. At the end of the growth curve, the knowledge of the massed curriculum students increased faster. In the last test, the massed curriculum students outperformed those in the spaced-out curriculum. The way students acquired and applied their knowledge was similar in both curricula. It seems, however, that students benefitted more from massed than spaced-out education, which may be due to the comprehensive integrated teaching involved.

## Introduction

Ensuring that medical students acquire and retain knowledge of oncology during their medical education is essential for excellent oncological care later on. Most doctors will face many oncological patients during their practice, regardless of their specialization. Potosky and colleagues (2014) [1] conducted a survey of US physicians showing that many physicians, without a specialization in oncology, lack critical knowledge and education on this topic. Moreover, these physicians lacked confidence in their knowledge of cancer. Similar results were found in undergraduate medical students [2]. To address this problem, several curricula for residency education were developed to provide structure, content, and guidance in teaching oncology [3]. Furthermore, integrated holistic approaches are increasingly being implemented in undergraduate oncology education [4]. Despite the number of curricula that have emerged, the efficacy of oncology education remains unclear [3]. In the Netherlands, cancer has been the leading cause of death since 2009. Since then, oncology has played an important role in the medical curriculum in undergraduate education. It is crucial that medical students acquire and retain knowledge of oncology during their preclinical phase, so they can apply that knowledge when face to face with a patient.

One way of improving students’ knowledge retention is by spacing and repeating the learning material throughout medical school. Spacing the study sessions improves long-term retention as compared to massed practice [5, 6]. In this study, we compared the influence of teaching oncology spread over a 3-year Bachelor’s phase with what is known as massed presentation (with most of the study material concentrated in one semester). Both curricula covered the same content, and both were used in the same medical school. We hypothesized that the spaced-out curriculum would prove more beneficial for students’ learning and retention, since students would re-study the learned topic throughout the Bachelor’s phase.

## Methods

### Setting

Since 2009, the University Medical Center Groningen (UMCG) has offered an international Bachelor’s degree program in medicine in parallel with a national Bachelor’s program. Both programs have the same learning goals, content, material, and teaching methods (PBL), but the order of disciplines is as different as the language used. At this stage, that is, the Bachelor’s phase, students do not have individual contact with real patients. During the patient lectures, patients are carefully selected. Most of these patients speak basic English, although in some cases the lecturer translates the answers given by the patient.

In the national track, the program is taught in Dutch, whereas in the international track, the program is taught in English.

Both groups have the same admission requirements. All international students take a proficiency in English test (IELTS) to ensure that they are proficient in English, unless they are native English speakers. Moreover, students need to show proof of their level of science education. If they fail to do so, they are allowed to attend 1 year of pre-university education. The proficiency of both tracks is comparable in terms of their knowledge, and they are both regulated by the same rules and cutoff scores.

### Oncology Education

Both curricula follow a comprehensive integrated curriculum, which is structured and part of the curriculum design. The content, learning objectives, material, number of hours, and the teachers are the same for the national and international tracks. In addition to language, the only difference is that, in the national track, oncology is condensed into one semester at the beginning of the third year, whereas in the international track, it is spread out over the three Bachelor’s years.

Throughout the Bachelor’s phase, medical students in both curricula, in addition to being exposed to patient cases during patient lectures, encounter patient problems in the form of assigments on paper. These patient cases might contain topics related to oncology. Students from both curricula encounter similar patient cases. Since in the spaced-out curriculum, students have various ontological topics spread out over the entire Bachelor’s phase, they may have acquired more knowledge about oncology as compared to those students in the massed curriculum.

### Progress Test

To measure students’ knowledge of oncology, we used their progress test results from 2009 to 2012. The Dutch progress test is based on the Dutch National Blueprint for the Medical Curriculum, and it aims to assess knowledge at the end of the curriculum level. The Dutch progress test is administered four times a year, and each test contains 200 multiple-choice questions on all subjects, including oncology. Furthermore, the questions can be divided into vignette questions, in which a patient case is presented, and knowledge questions without any patient cases. The questions differ for each test; the level of difficulty, however, is the same for all progress tests.

The progress test has a formative and a summative format. Students cannot fail because of one test or one subject. After completing the progress test, students receive feedback per discipline, which compares their mean with the overall mean of their reference group, and which is thus an indication of their performance. In addition, they receive a fail, pass, or good grade. Over a 1-year period, students are required to pass three of the four progress tests; otherwise, they will have to repeat the progress test as a whole and not necessarily just the block that they failed (for more information about the Dutch progress test, see Tio et al. 2016 [7]).

The national track takes the test in Dutch, whereas the international track takes the test in English. The English test is translated by an official certified translator (native speaker) with years of experience in translating medical documents (including tests). This translation is revised by a native English-speaking physician. After that, the translation process is reviewed by members of the Board of Examiners of our university.

During the Bachelor’s phase, students from both curricula have to take 12 progress tests in total. These tests contain the same questions and are taken at the same time for both curricula.

### Data Analysis

The 2400 questions from the 12 progress tests were categorized, and so we were able to select all questions related to oncology (*n* = 185).

To analyze the growth of students’ knowledge of oncology, we used a mixed effect model with maximum likelihood (ML) estimation [8]. We chose this analysis because it handles the unbalanced sample and missing data [9]. Furthermore, this method allowed us to determine the shape of the growth curves (linear, quadratic, or cubic) and examined the effects of covariates (type of curriculum) on the growth curves. Time was categorized in months. Students in the massed group were coded as −1 and those in the spaced-out group as 1.

Several models were tested. We used a backward elimination starting with the saturated model to first assess the covariance structure (unstructured, compound symmetry, and first-order autoregressive) of the model. We then eliminated non-significant parameters. The models were compared using −2 log likelihood, Akaike’s Information Criterion (AIC), and Schwarz’s Bayesian Criterion (BIC) criteria; the smaller the statistical values, the better the model fits the data. By using this methodology, our outcomes were protected against a type I error, which means detecting an effect that is not present. However, protecting against a type I error increases the chances of finding a type II error, which means not detecting an effect even if it is present. From a practical point of view, the probability of finding a significant outcome is lower with a type II error—only a strong effect would result in a significant difference. Analyses were performed using the mixed model procedure in SPSS 23.0 statistical software.

## Results

Data from 525 medical students were retrieved. Of those, 436 followed the massed curriculum, and the remaining 89 students followed the spaced-out curriculum. Of the latter, 37% were Dutch, 20% European, 38% from the Middle East, and 5% from other countries. In the massed curriculum, the majority of the students were Dutch, although some of them were born in another country or had a different cultural background.

A cubic growth model with an unstructured covariance matrix fitted our data best. Students in both curricula showed an increase in knowledge of oncology. The type of curriculum was a significant predictor of the initial status, and linear, quadratic, and cubic slopes (Table 1).

**Table 1:** Results of cubic growth model with unstructured covariance matrix

Parameter	Estimation of the growth model
Intercept	9.699705*
Linear	−1.343423*
Quadratic	0.142924*
Cubic	−0.002512*
Group	2.560432*
Linear × group	0.219179*
Quadratic × group	−0.013840*

*p > 0.005

**Fig. 1:**
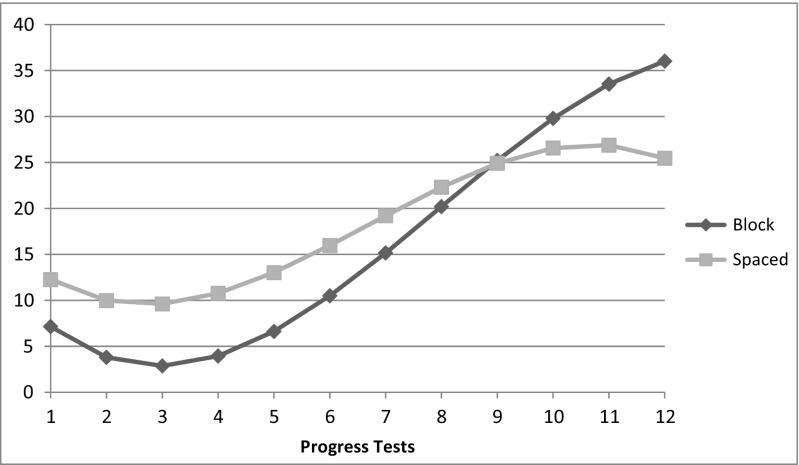
Growth trajectories of the massed group and spaced participants. Mean of the percentage of the correctly answered questions in oncology regarding the 12 progress tests that students took during their Bachelor phase

Initially, students in the spaced-out curriculum scored higher on oncology questions. The initial growth was faster for the spaced-out curriculum, whereas the acceleration over time was slower compared to the massed curriculum. At the end of the growth curve, the acceleration of the massed curriculum increased faster. In the four last tests, the massed curriculum outperformed the spaced-out curriculum (Fig. 1).

## Discussion

In this study, we hypothesized that students in a spaced-out curriculum would retain more knowledge of oncology than students in a massed curriculum. However, at end level, we found that students in the massed curriculum scored higher than students in the spaced-out curriculum.

Our finding is not in line with the spacing effect literature, which states that students in a spaced-out situation will score higher on a retention test than students in a massed situation [5, 6]. One explanation for our finding might be due to the comprehensive integrated teaching in our university. When students take the oncology block, they have already acquired basic knowledge of cancer, such as cell biology, and have solved a small number of cases. Students in the massed situation build up their basic knowledge of cancer before facing cases that require applying their knowledge, whereas students in the spaced-out curriculum had to apply their cancer knowledge before they had acquired the necessary basic knowledge. We have previously shown that medical students need to acquire basic factual knowledge first before they are able to apply their knowledge [10]. Alternatively, the massed curriculum is more structured than the spaced-out curriculum. In this case, structure refers to integrating the newly acquired knowledge with the existing knowledge [11], since the content is not spread out over a long period of time. The space between the oncology teaching moments for the spaced-out curriculum might be too far apart, which would hamper students’ performance on a retention test later on. Laboratory studies for simple knowledge have shown that the optimal gap between re-study sessions should be around 10–15% of the desired retention interval [5]. One implication might be that the effect of spacing oncology education may only become evident after a longer period of time.

Our study has a few limitations. First, the unbalanced sample between both curricula might have influenced the outcomes, despite the fact that we used an analysis method that deals with unbalanced samples. Second, there might be an influence from the language. The massed curriculum was taught in Dutch, whereas the spaced-out curriculum was taught in English. Both curricula answered identical questions in the progress tests, but the questions for the spaced-out curriculum were in English. The translation of the questions was a thoroughgoing process, which was supervised by an official translator, a native English-speaking medical doctor, the national progress test committee, and the Board of Examiners. Our retrospective study does not allow us to control for many of the variables that might have influenced our findings. However, the naturalist setting offers a closer look at real-life situations than laboratory research would, which is rarely possible in medical education. Another limitation may be the use of the Dutch progress test results, which measure knowledge at end level. However, the use of the progress test eliminates the bias of students’ willingness to participate, because it is mandatory. Furthermore, the progress test is a valid and reliable tool to measure students’ knowledge [12], whereas block tests are often not reliable or validated. Finally, we cannot determine whether massed education would retain more knowledge of oncology than spaced-out education after the Bachelor’s phase (i.e., 12 tests), because data from the clinical phase is not available.

Our study reveals that, after students have acquired basic factual knowledge of cancer, they might benefit more from studying oncology in a massed as opposed to a spaced-out curriculum. Further research should investigate whether spaced-out education would improve students’ performance in the clinical phase. Additionally, further studies should explore the space between re-studying sessions, which play a major role in improving long-term retention. Finally, further research needs to be conducted on different types of curricula, since a more traditional one might not repeat the same content as often as a problem-based learning curriculum.

## Conclusions

Curricula have a substantial impact on knowledge acquisition. The way students acquired their knowledge was similar in both curricula. It seems, however, that students did benefit more from massed education than from spaced-out education, which may be due to the comprehensive integrated teaching used.
